# The corpus callosum as anatomical marker of intelligence? A critical examination in a large-scale developmental study

**DOI:** 10.1007/s00429-017-1493-0

**Published:** 2017-08-11

**Authors:** René Westerhausen, Charline-Marie Friesen, Darius A. Rohani, Stine K. Krogsrud, Christian K. Tamnes, Jon S. Skranes, Asta K. Håberg, Anders M. Fjell, Kristine B. Walhovd

**Affiliations:** 1 Department of Psychology, Center for Lifespan Changes in Brain and Cognition (LCBC), University of Oslo, Blindern, POB 1094, 0317 Oslo, Norway; 2 Department of Laboratory Medicine, Children’s and Women’s Health, Norwegian University of Science and Technology, Trondheim, Norway; 3 Department of Medical Imaging, St. Olav’s Hospital, Trondheim, Norway; 4 Department of Neuroscience, Norwegian University of Science and Technology (NTNU), Trondheim, Norway; 5 Department of Radiology and Nuclear Medicine, Oslo University Hospital, Oslo, Norway

**Keywords:** Corpus callosum, Intelligence, Development, Structure–function association, MRI, Longitudinal studies

## Abstract

Intellectual abilities are supported by a large-scale fronto-parietal brain network distributed across both cerebral hemispheres. This bihemispheric network suggests a functional relevance of inter-hemispheric coordination, a notion which is supported by a series of recent structural magnetic resonance imaging (MRI) studies demonstrating correlations between intelligence scores (IQ) and corpus-callosum anatomy. However, these studies also reveal an age-related dissociation: mostly positive associations are reported in adult samples, while negative associations are found in developing samples. In the present study, we re-examine the association between corpus callosum and intelligence measures in a large (734 datasets from 495 participants) developmental mixed cross-sectional and longitudinal sample (6.4–21.9 years) using raw test scores rather than deviation IQ measures to account for the ongoing cognitive development in this age period. Analyzing mid-sagittal measures of regional callosal thickness, a positive association in the splenium of the corpus callosum was found for both verbal and performance raw test scores. This association was not present when the participants’ age was considered in the analysis. Thus, we did not reveal any association that cannot be explained by a temporal co-occurrence of overall developmental trends in intellectual abilities and corpus callosum maturation in the present developing sample.

**Electronic supplementary material:**

The online version of this article (doi:10.1007/s00429-017-1493-0) contains supplementary material, which is available to authorized users.

## Introduction

Intellectual and general cognitive abilities are supported by a large-scale brain network encompassing association cortices in frontal, parietal, and temporal lobes (Deary 2012; Deary et al. 2010; Jung and Haier 2007; Luders et al. 2009; Shaw 2007). The nodes constituting this network are distributed across both cerebral hemispheres, emphasizing the relevance of functional interaction between the hemispheres for performance in tasks demanding higher intellectual abilities (e.g., Belger and Banich 1998; Davis and Cabeza 2015; Welcome and Chiarello 2008, for review see Banich 2003). Consequently, it has long been suggested that the strength of the commissural connections—e.g., reflected in the midsagittal size of the corpus callosum—might serve as an anatomical marker of higher intellectual abilities (Hulshoff Pol et al. 2006; Men et al. 2014; Spitzka 1907; Strauss et al. 1994). A larger corpus callosum and/or thicker myelinated callosal axons would improve inter-hemispheric connectivity and, in turn, intellectual performance. In line with this interpretation, a common genetic origin for corpus callosum size and intelligence has been suggested in studies on healthy twins and siblings (Hulshoff Pol et al. 2006). This general notion is supported by a series of recent magnetic resonance imaging (MRI) studies demonstrating correlations between intelligence coefficients (IQ; as measured with standard intelligence tests) and measures of mid-sagittal callosal area (e.g., Allin et al. 2007; Ganjavi et al. 2011; Hutchinson et al. 2009; Peterson et al. 2001), thickness (Luders et al. 2007, 2011), and microstructural integrity (i.e., fractional anisotropy; Chiang et al. 2009; Dunst et al. 2014; Hutchinson et al. 2009; Navas-Sanchez et al. 2014; Tang et al. 2010). These studies also reveal an apparent dissociation in the direction of the reported correlations as a function of age. Studies examining adult samples mostly report positive associations (Chiang et al. 2009; Dunst et al. 2014; Luders et al. 2007; Strauss et al. 1994; and selectively in female participants in Tang et al. 2010). On the other hand, studies examining children, adolescents, and young adults report negative associations (Allin et al. 2007; Ganjavi et al. 2011; Hutchinson et al. 2009; Luders et al. 2011; but see Nosarti et al. 2004). For example, Luders et al. (2007) studying a mostly adult sample (age range 16–44 years) observed significant positive correlations between IQ measures and thickness in the posterior half of the corpus callosum. Studying a developing sample (age range 6–17 years) with the same methodological approach, the same group found a significant negative correlations, again located in the posterior corpus callosum (Luders et al. 2011). It has been suggested that this developmental dissociation might be attributed to differences in the relative weight of intra- vs. inter-hemispheric processing (Ganjavi et al. 2011) and age-related changes in task demands (Hutchinson et al. 2009).

When interpreting these previous findings it also has to be considered that in all the above studies intelligence was quantified using age-standardized, norm-deviation IQ scores. These deviation IQ scores reflect the relative position within the norm group, and do not represent the absolute level of performance (Angoff 1984; Neisser 1997; Wechsler 1999). As a consequence differences in performance levels between norm groups (i.e., between participants converted with different conversion tables) are removed and set to the norm distribution’s mean (usually 100; see e.g., Angoff 1984; Neisser 1997). The latter effect of the IQ conversion is especially pronounced when studying development samples since the provided conversion tables for children and adolescents cover very narrow age spans reflecting the rapid absolute intellectual development in this period of life. For example, the Wechsler Abbreviated Scale of Intelligence (WASI, Wechsler 1999), which was used by all of the above-mentioned developmental studies, provides conversion tables covering age spans of only 4 months for participants under the age of 16 year. As a result, the mean of the converted IQ scores will appear stable throughout childhood and adolescence (e.g., Burgaleta et al. 2014) although the absolute level of performance (e.g., the mean raw test scores) rises continuously in this period of life (Neisser 1997; Tamnes et al. 2010). While the conversion to IQ score allows evaluating age-appropriateness of an individual’s intellectual abilities and rank stability within a cohort over time, only the use of raw test score will allow to study the development of intellectual functioning. Therefore, we here argue that to address the question of whether corpus-callosum morphology can serve as marker of intellectual and cognitive abilities during development, measures of absolute rather than norm-relative performance should be used.

The present study was designed to systematically re-examine the association of IQ test measures and corpus-callosum anatomy during development in a large mixed cross-sectional and longitudinal sample (734 datasets) by for the first time using raw test scores rather than deviation IQ. This was done in three analyses steps. First, to establish a general structure–function association, we related verbal raw test scores (v-RS) and performance raw test scores (p-RS) to regional callosal thickness measures. As brain size has previously been found to be positively correlated with both corpus callosum size (Ganjavi et al. 2011; Jäncke et al. 1999; Westerhausen et al. 2016) and intelligence measures (for a meta-analyses see McDaniel 2005; or Pietschnig et al. 2015) total intracranial volume (TIV) was included as an additional predictor in a second analysis step. Finally, in a third step, we examined the effect of chronological age on the structure–function association. This was necessary as raw intelligence test scores (Neisser 1997; Tamnes et al. 2010) and callosal thickness (e.g., Giedd et al. 1996; Luders et al. 2010b; Westerhausen et al. 2016, 2011) are characterized by a continuous age-related increase in the studied age period, and the temporal co-occurrence of these two developmental trends could confound the association (see e.g. Salthouse 2011).

## Methods

### Participants

Participants were drawn from two longitudinal imaging studies, the Norwegian Mother and Child Cohort Neurocognitive study (NeuroCogMoBa; e.g., Krogsrud et al. 2016) and Neurocognitive Development (NeuroCogDev; e.g., Tamnes et al. 2013), both coordinated by the Lifespan Changes in Brain and Cognition (LCBC) research center, University of Oslo, Norway. The analyzed sample included 734 datasets from 495 (250 female) participants, of which 239 (131 female) provided measures for a second time point, and covering an age range between 6.4 and 21.9 years. This final sample represented a subsample of a total of 1085 data sets of which 83 data/participants were excluded due to a history of mental or neurological disorders, reported (by parents) to have premature birth (<37 weeks) and low birth weight (<2500 g), as well as due to low-quality of the T1-weighted MRI data. Another 263 datasets/participants had to be excluded as no WASI data was available or the available test data was incomplete. Thus, the final sample included all datasets of the above studies for which both high-quality MRI data and valid intelligence testing with the WASI were available.

The studies were approved by the Regional Committee for Medical and Health Research Ethics. Until the age of 16, the care legal guardians of the participants provided written informed consent. Additionally, informed assent was given in written form by participants above the age of 12, and in oral form by participants below the age of 12.

### Intelligence assessment

Intelligence assessment for all participants and time points was conducted with a Norwegian version of the WASI (Wechsler 1999), including the subtests Vocabulary and Similarities for estimating verbal abilities as well as the subtests Block-Design and Matrix Reasoning for estimating performance (non-verbal) abilities. Raw subscale (RS) scores were calculated as weighted average raw test score, that is, the proportion of correct answers (i.e., the individual score divided by the maximum score that could be achieved in that subtest) was calculated per subtest and averaged across the relevant verbal (v-RS), performance (p-RS), or all four subtests (fs-RS), respectively. For comparison, age-standardized deviation IQ scores for verbal (v-IQ), performance (p-IQ), and estimated “full-scale” IQ (fs-IQ) were obtained using the age-appropriate conversion tables provided in the WASI test manual.

For illustration, we performed an explorative regression analysis determining the empirical association between RS and standardized IQ measures. Both linear and quadratic effects of RS on IQ measures were included in the model. This analysis was done separately for participants tested the first and second time, respectively. Although all models were statistically significant (all *p* < 0.001), the association between RS and IQ scores was, as expected, far from perfect, explaining at maximum 42% of the variance. In detail, at baseline testing (*n* = 495) coefficient of determination was *R* ^2^ = 0.37 for the prediction of v-IQ, *R* ^2^ = 0.35 for the prediction of p-IQ, and *R* ^2^ = 0.31 for the prediction of fs-IQ. At follow-up testing (*n* = 239), it was *R* ^2^ = 0.26 for v-IQ, *R* ^2^ = 0.42 for p-IQ, and *R* ^2^ = 0.31 for the prediction of fs-IQ. Across all analyses both the linear (all regression weights positive) and quadratic predictors (all negative regression weights) were significant (all *p* < 0.05), except for the v-RS analysis at follow-up testing in which the quadratic prediction was not significant (*p* = 0.41). This overall pattern of association underlines the expected non-equivalence of RS and deviation IQ in a developmental sample (Neisser 1997). Thus, in accordance with the objectives of the present study we focused on the raw test scores for further analyses.

**Fig. 1:**
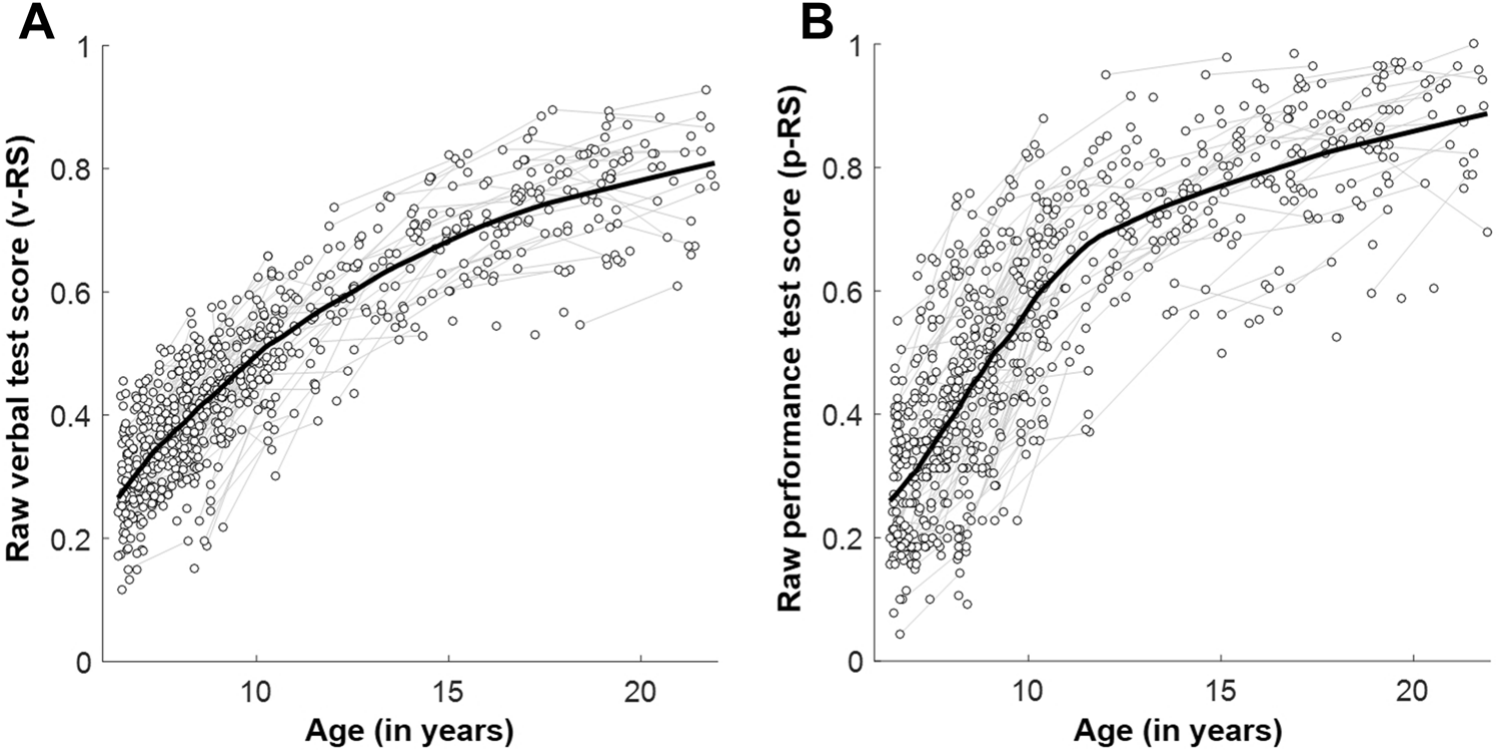
Intellectual development. Spaghetti plot illustrating the development of **a** raw verbal (v-RS) and **b** raw performance (p-RS) test scores for 495 participants of which 239 provided measures for two time points (connected with *gray lines*). *Solid black line* represents the locally weighted scatterplot smoothing (LOWESS, smoothing factor, *f* = 0.25)

To further describe the developmental trajectories of v-RS and p-RS, we also conducted two separate linear-mixed model analyses (restricted maximum likelihood estimations, REML) using chronological age as linear (Age) and quadratic (Age squared) predictors (fixed effects), as well as allowing for different intercepts between participants (random effect term). In both cases, Age (v-RS: *β*_Age_ = 0.049; *t*_731_ = 47.3, *p* < 0.0001; p-RS: *β*_Age_ = 0.061; *t*_731_ = 37.8, *p* < 0.0001) and Age-squared (v-RS: *β*_Age squared_ = −0.0025; *t*_731_ = −14.4, *p* < 0.0001; p-RS: *β*_Age squared_ = −0.0036; *t*_731_ = −13.8, *p* < 0.0001) contributed significantly to the prediction, together describing the monotonous increase of level of performance which flattens towards young adulthood (see Fig. 1).

### MRI acquisition

The MRI data used in the present analysis stems from two different research projects (NeuroCogMoBa, NeuroCogDev) and two different sites (Rikshospitalet, Oslo, and St. Olav’s Hospital, Trondheim), but all MRI scans were acquired with the same scanner model and equipment (1.5T Siemens Avanto, a 12-channel head coil) as well as using the very same pulse sequences. T1 weighted images were acquired with the following parameters: echo time, TE = 3.61 ms, repetition time, TR = 2400 ms, inversion time, TI = 1000 ms, and a flip angle of 8 degrees. For each participant 160 sagittal slices with a thickness of 1.2 mm were taken, with an image resolution of 1.25 × 1.25 × 1.2 mm^3^, a field view of 240 × 240 mm^2^ and a 192 × 192 scan matrix. The data acquisition for the MOBA participants was done using a parallel imaging technique (iPAT, GRAPPA factor 2) acquiring multiple (between 2 and 4) T1 volumes in a short scan time (4 min and 18 s per volume). Of the final 734 datasets 542 datasets were acquired in Oslo and 192 in Trondheim. Raw image quality was assessed based on visual inspection and performed by two experienced examiners (D.A.R., S.K.K.) and the best dataset was used for the analysis.

### Measurement of callosal thickness

Corpus callosum morphology was assessed using thickness measurements determined on callosal outline on the mid-sagittal cross-sectional surface area on the T1-weighted images. This approach was preferred over the traditional geometrical subdivision since it is less dependent on inter-individual differences in the curvature of the corpus callosum and offers a better regional specificity (Luders et al. 2006). The present implementation of the approach has been described elsewhere (Westerhausen et al. 2016), and is briefly summarized here. First, all individual images were coregistered to a template using a rigid-body transformation and segmented in native space (using SPM12; http://www.fil.ion.ucl.ac.uk/spm/). Then, in a semi-automated procedure, the corpus callosum was identified on the midsagittal slice of the segmented white-matter volume and manually adjusted where necessary (to remove “non-callosal” voxels, e.g., fornix). The resulting callosal mask was extracted as binary image and an outline was created by removing all non-border voxels from the segmented corpus callosum. The tip of the rostrum (posterior-most voxel of the in-bend rostrum in the anterior half) and base of the splenium were identified (ventral-most voxel in the posterior half). Subsequently, the outline was rotated so that the imagined line connecting rostrum tip and base of the splenium were horizontally oriented. Rostrum tip and the base of the splenium determined the division into a ventral (“lower”) and dorsal (“upper”) outline. The midline between ventral and dorsal outline was determined based on support points spaced equidistantly on the two outlines. Finally, the midline was resampled into 60 equidistant points along the midline, orthogonal to which callosal thickness was determined as distance between ventral and dorsal outline. The resulting 60 regional thickness measures for each participant and time point were used for the statistical analysis.

### Measurement of brain compartment volume

Total intracranial volume, TIV, estimates were obtained by means of automated segmentation routines (“tissue volumes” utility) using SPM12, and was defined as the sum of grey matter, white matter, and CSF compartments, including forebrain, midbrain, hindbrain, and cerebellum. The inferior limit for TIV estimation was set at the boarder of the cerebellum. This approach was selected as it has demonstrated high external validity (Hansen et al. 2015). The mean TIV was 1.43 L (SD = 0.14) across all subjects and time points, ranging from 1.10 to 2.01 L.

### Statistical analysis

To integrate cross-sectional and longitudinal data in the same analysis, a linear mixed model design (Verbeke and Molenberghs 2009) was conducted for the regional thickness analysis. To establish a general association between RS intelligence measures and callosal thickness, the first set of analyses (step 1) included a fixed-effect part with the predictors for the respective Test Score (either v-RS, or p-RS; demeaned continuous variable), Sex, as well as the interaction of these two variables. The random effect part consisted of Participant (allowing for different intercepts) to account for the repeated measures available for 239 of the participants. Additionally, handedness (coded as right-handed vs. non-right-handed) and Site were added as nuisance variables. Handedness was included as it is related to differences in corpus callosum size and thickness (Luders et al. 2010a; Westerhausen et al. 2004). The predictor Site was included to account for possible differences resulting from using different MR scanners, an approach which can be considered appropriate as the here aggregated studies were conducted on the same scanning platforms and used the same scanning sequence (Chen et al. 2014). The regional thickness in each of the 60 segments, served as dependent variable. In analysis step 2, the above model was extended by introducing TIV as additional covariate. In step 3, chronological age was considered by adding predictors representing linear (Age) and quadratic relations (Age squared), as well as the interaction of Test Score and Age. The same three-step analysis was conducted using the fs-RS. However, as fs-RS was naturally highly correlated with the two constituting sub-test scores (see section Intelligence Assessment), we here only present analyses for v-RS and p-RS in detail (see Supplement Fig. 1 for the analyses of the fs-RS data). Although the use of deviation-IQ scores was not considered appropriate for the present research question the three analysis steps were also conducted for the respective IQ scores (v-IQ, p-IQ, fs-IQ) to allow the interested reader to compare the present to previous findings. The results of these analyses are provided in Supplement Fig. 2.

All analyses were conducted utilizing restricted maximum likelihood estimations (using a full covariance matrix; Cholesky parameterization), and the models were fitted with the “fitlme” function provided in MATLAB (R2015b, The MathWorks, Natick, MA, USA). The significance level was adjusted to a false-discovery-rate (FDR) of 0.05 using Benjamini–Hofberg–Yekutieli (Benjamini and Yekutieli 2001) procedure (i.e., not assuming positive dependency of the tests within one analysis). The results are presented with unstandardized regression weights (β) to express the effect on callosal thickness in absolute measures (i.e., mm) as well as using percentage explained variance (*ω* ^2^) as relative measure.

Across analyses, the effects of interest were: (a) the main effect of Test Score (v-RS or p-RS), indicating an overall structure–function association (analysis steps 1–3); (b) the interaction of Test Score with Sex, indicative of slope difference between male and female subjects (step 1–3); (c) the interaction of Test Score with Age, indicative of changes in the slope during development (step 3). For the effects of interest, the main analyses were supplemented with a power analysis using G*Power 3 (Faul et al. 2009), whereby a sensitivity analysis was conducted to determine the smallest effect size which can be detected with a power of 0.80 and 0.95. The power analysis was calculated assuming an *α*-level of 0.00083 (i.e., representing a scenario with FDR threshold reaching Bonferroni threshold for 60 tests), two-tailed testing, and considering the fixed-effect part only. The smallest detectable effect size was Cohen’s *f* ^2^ = 0.024 and *f* ^2^ = 0.034, for 0.80 and 0.95 test power, respectively (only marginally differing from step 1 to step 3, with 5–9 predictors, respectively) which is equivalent to an explained variance of *ω* ^2^ = 0.023–0.033. That is, population effects larger than 2.3 or 3.3% can be excluded for non-significant associations.

## Results

**Fig. 2:**
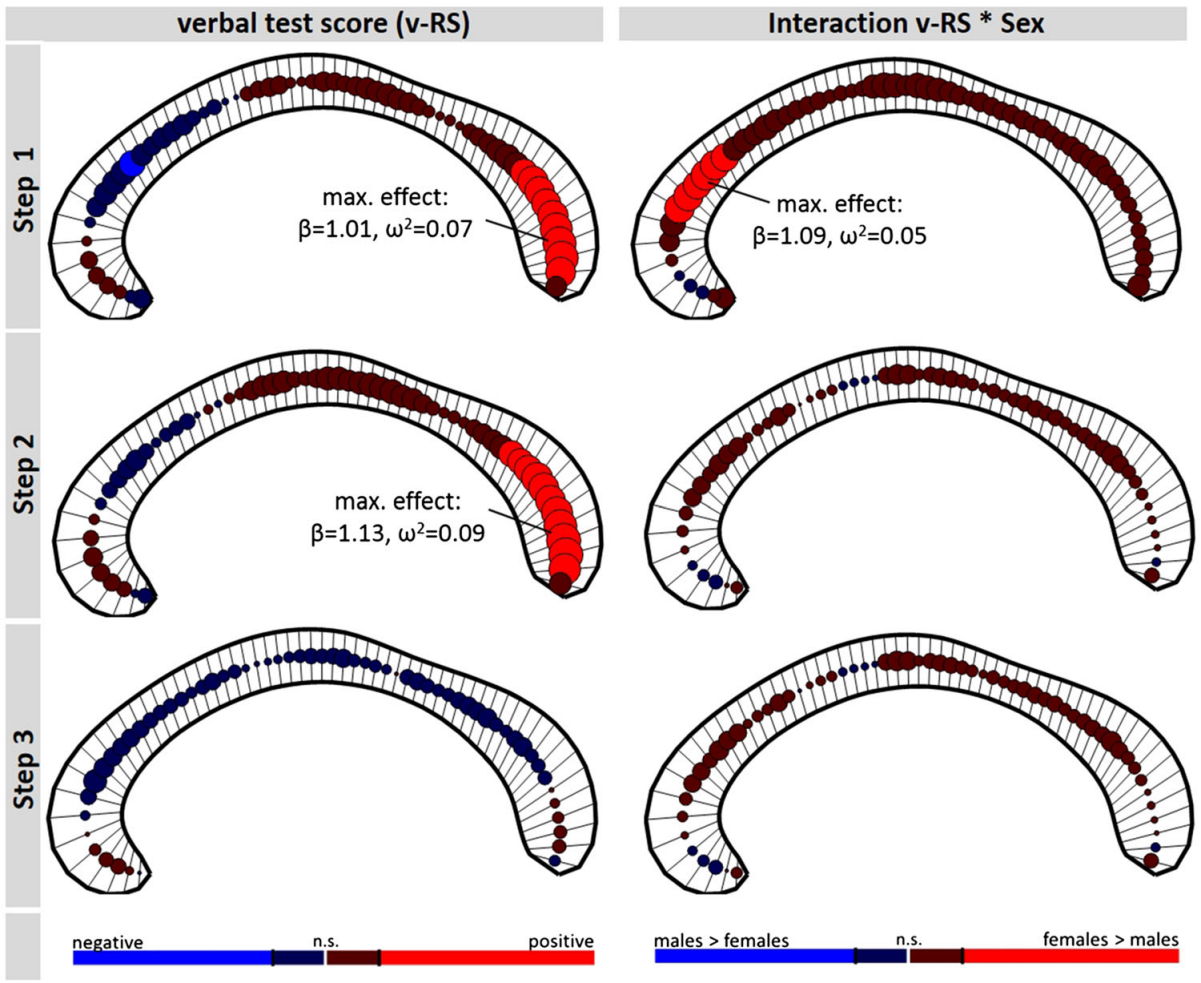
Association raw verbal test score (v-RS) and regional callosal thickness (*left column*) and the interaction of v-RS with Sex (*right column*). The *rows* represent the three analysis steps (full models described in “Method” section), with the statistical design of step 2 compared to step 1 additionally including TIV as covariate, and step 3 compared to step 2 additionally including age-related variables (i.e., Age linear, Age squared, and the interaction of v-RS and Age). At each of the 60 segments of the corpus callosum, the direction and magnitude of the association is visualized by a *circle*, whereby the size of the *circle* is proportional to empirical *t* value and the *color*, *red* vs *blue*, codes positive and negative associations, respectively. *Lighter red* and *lighter blue* indicate significant associations, with the significance level being adjusted to a false-discovery-rate (FDR) of 0.05. The anterior corpus callosum is on the *left side* of each *panel*

**Fig. 3:**
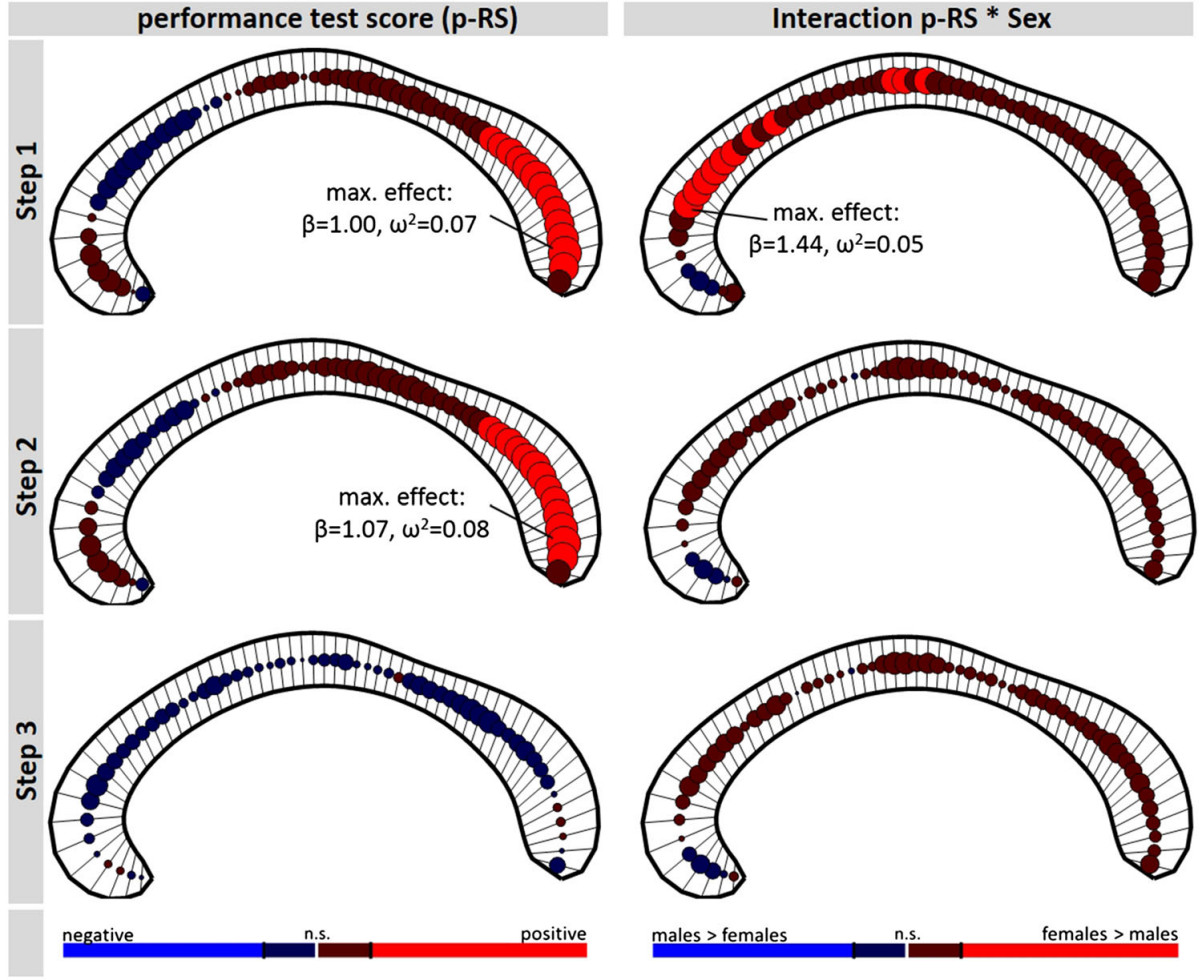
Association of raw performance test score (p-RS) and of regional callosal thickness (*left column*) and the interaction of p-RS with Sex (*right column*). As in Fig. 2, *rows* represent the three analysis steps. At each callosal segment, the direction and magnitude of the association is visualized by a *circle*. The size of the *circle* is proportional to empirical *t* value and positive and negative associations are coded *red* and *blue* respectively. *Lighter red* and *lighter blue* indicate significant associations [significance level adjusted to a false-discovery-rate (FDR) of 0.05]. The anterior corpus callosum is on the *left side* of each *panel*

**Fig. 4:**
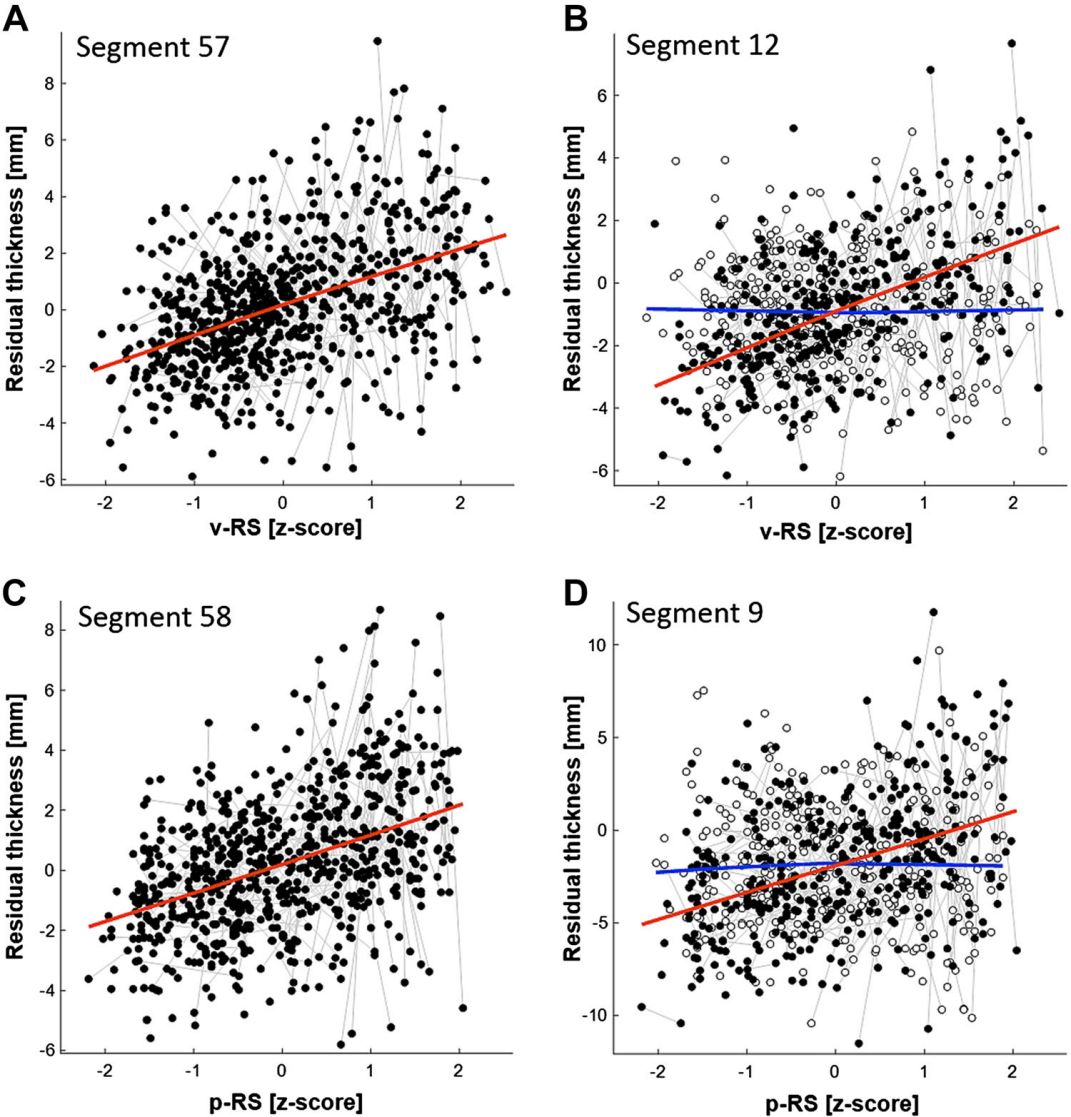
Spaghetti plots at location of maximum effect of analysis step 1. Plot **a** shows the main effect of verbal raw (v-RS) test scores on callosal thickness (residualized for all other effects) in segment 57 (*red line*). Plot **b** illustrates the interaction of v-RS with Sex with *filled circles* representing female (*red line*) and *open circles* representing male (*blue line*) participants. Plot **c** illustrates the main effect of performance raw (p-RS) test scores on residualized callosal thickness in segment 58 (*red line*). Plot **d** shows the interaction of p-RS with Sex with *filled circles* representing female (*red line*) and *open circles* representing male (*blue line*) participants

The analysis 1 for both p-RS and v-RS revealed a significant positive association of test performance and callosal thickness in the posterior corpus callosum including most parts of the splenium section (see upper row, left panel, Figs. 2, 3). For p-RS the strongest association was found in segment 58 with an explained variance of *ω* ^2^ = 0.07 (*β*_p-RS_ = 1.00; *t*_728_ = 5.01, *p* < 0.0001, see Fig. 4). Comparably, for v-RS the strongest association was also found in segment 57 (*β*_v-RS_ = 1.01; *t*_728_ = 5.03, *p* < 0.0001; *ω* ^2^ = 0.07, see Fig. 4). The v-RS analysis additionally revealed a negative association in the genu of the corpus callosum, that is, in segment 13 (*β*_v-RS_ = −0.55; *t*_728_ = −3.14, *p* = 0.0018, *ω* ^2^ = 0.03, see Fig. 2). In addition to the main effect, both analyses also revealed a significant interaction of test score and Sex indicating differences in the slope of the association between the sexes. In the p-RS analysis the interaction was found in extended areas in the genu as well as in the truncus region with the maximum effect being located in segment 9 (*β*_p-RS*Sex_ = 1.44; *t*_728_ = 4.12, *p* < 0.0001, *ω* ^2^ = 0.05). Across all significant segments, the interaction was driven by a more positive slope in female than in male participants (see Fig. 4d). In the v-RS analysis the area of significant interactions was restricted to the genu region with the maximum being located in segment 12 (*β*_v-RS*Sex_ = 1.09; *t*_728_ = 4.17, *p* < 0.0001, *ω* ^2^ = 0.05). Also here the interaction was driven by a more positive slope in female than male participants (see Fig. 4b).

**Fig. 5:**
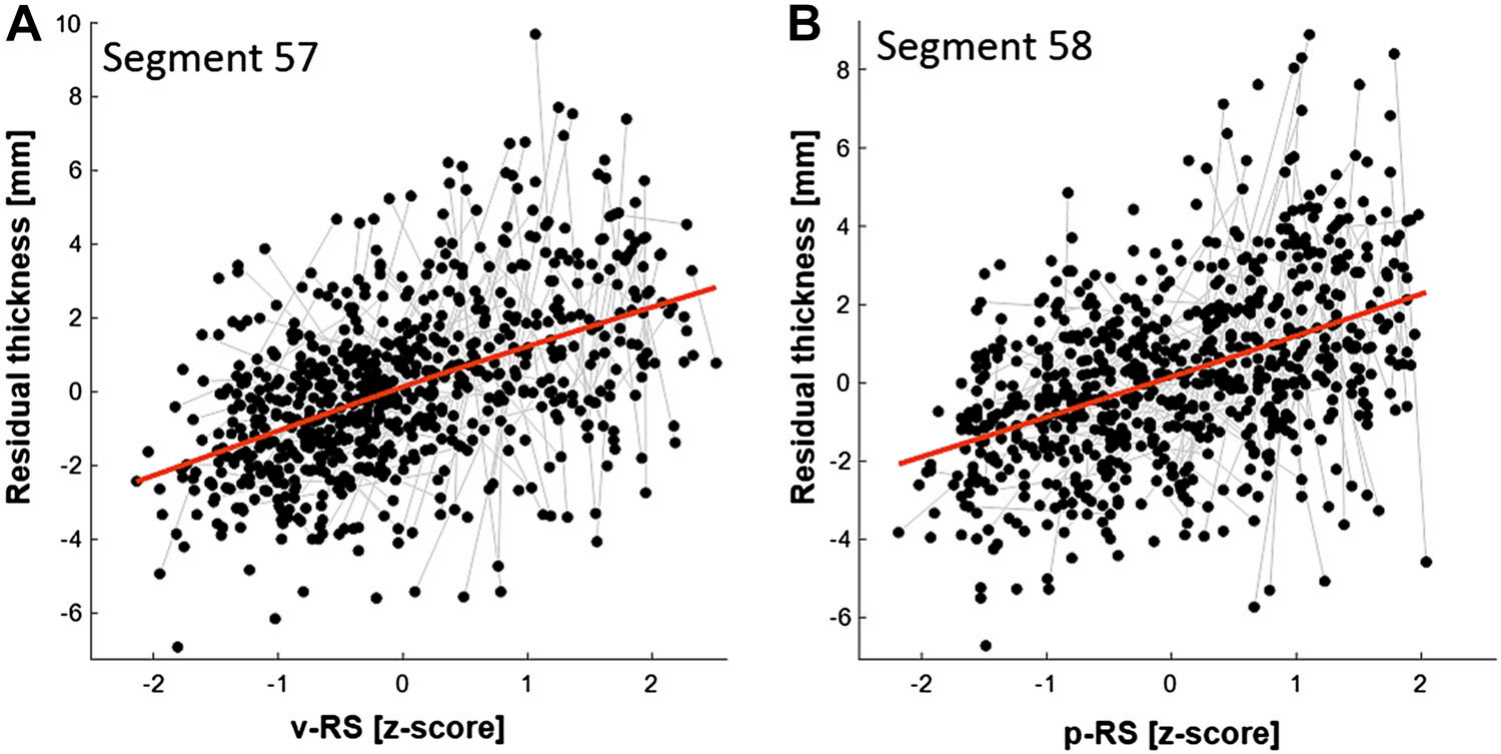
Spaghetti plots at location of maximum effect of analysis step 2. Plot **a** illustrates the main effect of verbal raw (v-RS) test scores on residualized callosal thickness in segment 57 (*red line* depicting linear fit) after considering TBV differences. Likewise, plot **b** shows the main effect of performance raw (p-RS) test scores on residualized callosal thickness in segment 58 (*red line* depicting linear fit)

In analysis step 2, including TIV as covariate, for both intelligence measures a positive association with thickness in posterior callosal segments was found (see Figs. 2, 3, second row). Comparable to analysis step 1, the strongest association was located in segment 57 for the v-RS analysis (*β*_v-RS_ = 1.13; *t*_727_ = 5.54, *p* < 0.0001; *ω* ^2^ = 0.09, see Fig. 5) and in segment 58 for the p-RS analysis (for p-RS: *β*_p-RS_ = 1.07; *t*_727_ = 5.36, *p* < 0.0001; *ω* ^2^ = 0.08). However, including TIV in the model, the interaction of Sex and Test Score was no longer significant (for v-RS all |*β*_v-RS*Sex_| < 0.51; all |*t*_727_| < 1.66; and for p-RS all |*β*_p-RS*Sex_| < 0.69; all |*t*_727_| < 2.08; all non-significant using FDR correction).

**Fig. 6:**
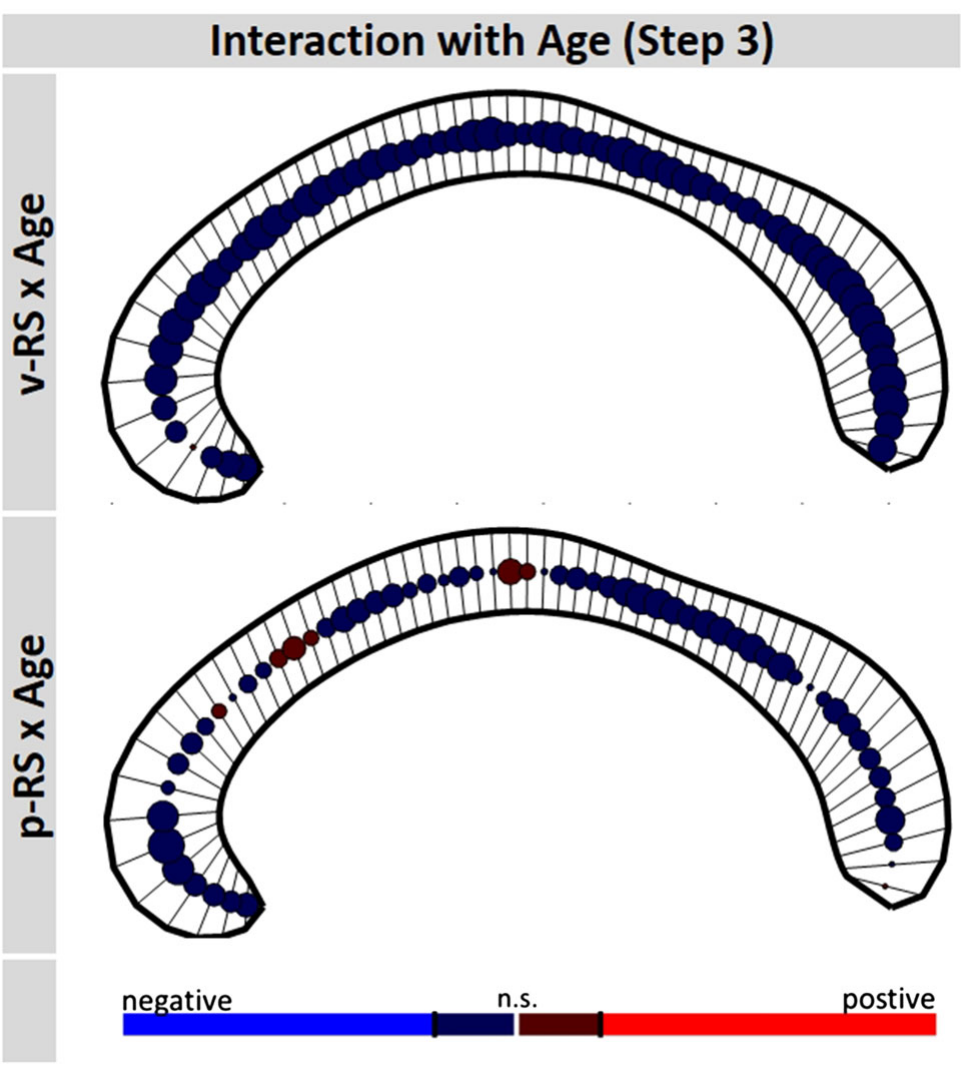
Interaction of raw test scores (performance, p-RS; verbal, v-RS) and Age in predicting regional callosal thickness in analysis step 3. At each callosal segment, the direction and magnitude of the association is visualized by a *circle*. The size of the *circle* is proportional to empirical *t* value of the predictor coding the interaction; positive and negative associations are coded *red* and *blue*, respectively. Significance level is adjusted to a false-discovery-rate (FDR) of 0.05. The anterior corpus callosum is on the *left side* of each *panel*

Finally, including Age covariates (i.e., linear term, quadratic term, interaction of test score and Age) in analysis step 3, the main effect of test score vanished for both test subscores (for v-RS all |*β*_v-RS_| < 1.04, all |*t*_724_| < 2.41; for p-RS all |*β*_p-RS_| < 0.76, all |*t*_724_| < 2.00; all non-significant using FDR correction) for both p-RS and v-RS analyses. In addition, the interaction of test score and Sex did not reach significance (for v-RS all |*β*_v-RS*Sex_ | < 0.47, all |*t*_724_| < 1.53; and for p-RS all |*β*_p-RS*Sex_| < 0.70, all |*t*_724_| < 2.11). In neither of the two analyses a significant interaction of Test Score and Age was found (for v-RS all |*β*_v-RS*Age_| < 0.20, all |*t*_724_| < 2.72; and for p-RS all |*β*_p-RS*Age_| < 0.22, all |*t*_724_| < 2.52; all non-significant using FDR correction, see Fig. 6), that is, the association of intelligence and callosal thickness was not significantly modulated by Age.

## Discussion

The present study re-examined the relationship between intellectual development and callosal maturation during childhood, adolescence, and young adulthood. When not accounting for chronological age, a positive association in the splenium of the corpus callosum was detected for both p-RS and v-RS. At the thickness segments with the largest effect size, an increase in p-RS or v-RS by one standard deviation was reflected by an increase in regional callosal thickness of about 1 mm. This positive association was found irrespective of whether individual differences in brain size were accounted for in the analysis or not. Considering the known topographical organization of the corpus callosum, the splenium region interconnects temporal, parietal, and occipital cortices, including sensory and higher association areas (Putnam et al. 2010; Schmahmann and Pandya 2006; Westerhausen et al. 2009). Temporal, parietal, and occipital regions have shown associations to IQ scores in developing (Burgaleta et al. 2014; Karama et al. 2011; Menary et al. 2013; Shaw et al. 2006) as well as adult samples (Choi et al. 2008; Haier et al. 2004; Narr et al. 2007). Furthermore, the present posterior callosal association might reflect especially the maturation of the “parietal functions” within parieto-frontal integration theory of intelligence (PFIT; Jung and Haier 2007), and be associated in particular with early processing and integration of sensory information. A “non-functional” interpretation is also conceivable; any callosal variability is likely to reflect differences in the developing cortical architecture but might not itself contribute to the level of performance (Strauss et al. 1994). That is, the corpus callosum would serve as a “marker” for an “intelligent brain”.

However, the above interpretation deserves further clarification. When introducing chronological age into the statistical model, the suggested associations between callosal thickness and raw intelligence scores disappeared. In the studied period of life, between 6 and 22 years, it is known that intelligence test performance when expressed as raw scores (Tamnes et al. 2010, see also Fig. 1) and callosal thickness (e.g., Giedd et al. 1996; Luders et al. 2010b; Westerhausen et al. 2016) are characterized by a continuous, monotonous age-related increase. Consequently, the temporal co-occurrence of these two developmental trends alone could drive the present structure–function association without the two variables necessarily being interrelated (e.g., Salthouse 2011, for a more general discussion). Thus, to examine the interdependence beyond the co-occurring age-related covariance, we introduced chronological age to the design, which yielded the effect of Test Score for both p-RS and v-RS non-significant. This “null” finding by itself does not unequivocally exclude the existence of a true functional association between intelligence measures and callosal thickness. Assuming, for example, that callosal maturation would be the only determinant of intelligence test performance, both variables would exhibit a strong covariation of rates of change during development, a correction for age would likely leave the two variables uncorrelated. However, the also non-significant interaction of Test Score and Age further indicates that the lack of covariance of Test Score and callosal thickness is invariant across all ages studied (Hofer et al. 2006; Salthouse 2011). Thus, the lack of a significant effect of Test Sore together with the non-significant interaction of Test Score and Age, indicate that for no chronological age a significant structure–function association was found. Given sufficient test power to exclude effects larger than 2–3% explained variance, the present findings render any substantial functional relevance of macrostructural callosal variability for intelligence test performance unlikely, within the studied age range.

The non-relevance of individual differences in callosal thickness for intelligence test performance is, on one hand, not surprising as it is in line with studies on patients with partial or complete callosotomy (e.g., Mamelak et al. 1993; Oguni et al. 1991; Tanriverdi et al. 2009). Pre-post-surgery comparisons usually fail to find a substantial decrease in IQ scores as consequence of the callosal transsection. For example, Mamelak et al. (1993) examined 15 epilepsy patients aged between 9 and 31 years (i.e., also covering the age range examined here) and did neither find substantial change in verbal (mean change in v-IQ: 0.38, SD = 3.52; from Table 3, p. 692, of Mamelak et al. 1993) nor in performance IQ (mean change in p-IQ: 1.23, SD = 3.56). Nevertheless, it has to be kept in mind that findings in callosotomy patients are not fully representative, as the patients’ pre-surgical brain anatomy certainly deviates from a healthy brain including plastic adaption processes to the epilepsy-related changes (Campbell et al. 1981). On the other hand, the present findings are partly at odds with a series of earlier studies demonstrating that inter-individual difference in callosal architecture are predictive of functional inter-hemispheric integration in tasks assessing cognitive-control functions (Davis and Cabeza 2015; Huster et al. 2011; Kompus et al. 2011; Schulte et al. 2006). However, a critical component of these tasks was that speeded choice reactions were required, demanding a rapid exchange of information between the hemispheres. The performance in the four tasks which constitute the WASI test, on the other hand, is not relying on fast stimulus–response mapping in a similar manner. Also the association between general white-matter tract fractional anisotropy and intelligence measures has been previously shown to be mediated by general information-processing speed (Penke et al. 2012). Thus, it is conceivable that in the present study no structure–function association was found since time-critical communication between the hemispheres was not required to the same extend in the WASI tests.

The present findings also need to be discussed in relation to previous studies correlating deviation IQ scores with measures of callosal anatomy in comparable developmental samples. Irrespective of whether interpreting the outcome of the analyses without (positive association) and with Age covariates (no association), the present findings do not align with a series of previous developmental studies reporting negative associations (Allin et al. 2007; Ganjavi et al. 2011; Hutchinson et al. 2009; Luders et al. 2011). As also argued in the introduction, deviation IQ scores reflect the relative position of an individual within a specific norm age group and no longer the absolute level of performance (Angoff 1984). This is, for example, reflected in the present sample as raw scores in the prediction of IQ scores explains at maximum 42% of the variance (see “Method” section). Furthermore, the conversion from raw measure to IQ score also removes overall differences in performance level between individuals that fall into different age groups (different conversion table are used) and set to the mean to the norm distribution’s mean (Wechsler 1999). The converted IQ scores will consequently appear stable (Burgaleta et al. 2014) throughout childhood and adolescence while the absolute level of performance naturally rises continuously in this life period (see Fig. 1). At the same time, however, none of the previous studies converted (or adjusted to norm-data) callosal morphology measures. Rather, two of the above developmental studies (Allin et al. 2007; Luders et al. 2011) related deviation IQ scores to raw measures of midsagittal callosal area and regional callosal thickness, respectively, both without correcting for chronological age. Considering the properties of deviation IQ the reported findings can be reformulated to: the participants’ relative position in performance with respect to their age-specific norm group was negatively associated with the participants’ absolute measures of corpus-callosum size/thickness. In the present study, a positive correlation between absolute level of performance and absolute callosal thickness was detected (when not considering Age in the analysis). Of note, the attempt to replicate the negative associations of Luders et al. (2011) and Allin et al. (2007) using deviation IQ scores did not yield any significant associations in the present sample (see Supplementary Fig. 2). Two other studies (Ganjavi et al. 2011; Hutchinson et al. 2009) introduced chronological age as covariate into the statistical design while at the same time keeping the already age-adjusted deviation IQ score. While the covariate will remove age-related variance also from the callosal measures by implicitly treating these as deviation from the predicted mean for the respective age, it does not express the data relative to an external norm distribution as it is done for deviation IQ score, again making the interpretation difficult. Considering Age as covariate and in interaction with Test Scores in the present study, did not reveal any significant association between test performance and absolute thickness.

Finally, previous studies also report sex differences in the association of callosal and deviation IQ measures (Dunst et al. 2014; Luders et al. 2007; Tang et al. 2010), whereby in developmental samples a more negative associations in male compared to female subsamples has been reported (Ganjavi et al. 2011; Luders et al. 2011). In the present study, we find a Test score by Sex interaction in the genu of the corpus callosum for v-RS and p-RS in analysis step 1, which was in both cases driven by a positive association in females while no significant association was found in male participants (see Fig. 4). As axons located in the genu interconnect prefrontal cortices (Benedictis et al. 2016; Schmahmann and Pandya 2006) the findings might relate to the frontal components of intelligence (Jung and Haier 2007), whereby selectively female participants seem to benefit from a stronger structural connectivity via the corpus callossum. However, this dissociation only holds for “absolute” callosal measures; once TIV was accounted for the interaction did not survive FDR correction.

In summary, intellectual abilities are supported by a large-scale bihemispheric network (Deary 2012; Jung and Haier 2007; Shaw 2007) suggesting functional relevance of inter-hemispheric coordination (Banich 2003; Davis and Cabeza 2015). The present study was able to confirm that a general structure–function correlation exists during development but only as long as the participants’ age was not considered. Thus, we did not find any association that cannot be explained by a temporal co-occurrence of overall developmental trends in intellectual development and structural callosal increase. However, we here examined the macrostructural development using regional thickness measures. It remains for future studies to determine whether these findings are confirmed when indices of microstructural differences, such as fractional anisotropy, are assessed. Future studies also have to establish, whether the present finding utilizing RS instead of deviation-IQ extend into adult age, as the structural development of the corpus callosum continuous at least to the end of the third life decade (e.g., Pujol et al. 1993).

## Electronic supplementary material

Below is the link to the electronic supplementary material.

Suppl. Figure 1. Association estimated full-scale raw test score (fs-RS) and of regional callosal thickness (left column) and the interaction of fs-RS with Sex (right column). The rows represent the 3 analysis steps (full model described in Method section), with the statistical design of step 2 compared to step 1 additionally including TIV as covariate, and step 3 compared to step 2 additionally including age-related variables (i.e., Age, Age squared, and the interaction of fs-RS and Age). At each of the 60 segments of the corpus callosum, the direction and magnitude of the association is visualized by a circle, whereby the size of the circle is proportional to empirical t value and the color (red vs. blue) codes positive and negative associations, respectively. Lighter red and lighter blue indicate significant associations, with the significance level is adjusted to a False-Discovery-Rate (FDR) of 0.05. Note: the anterior corpus callosum is on the left side of each panel. (JPEG 410 kb)

Suppl. Figure 2. Association of verbal (v-IQ, left column), performance (p-IQ, middle column), and estimated full-scale (fs-IQ, right column) deviation IQ with regional callosal thickness. The rows represent three analysis steps (equivalent to analysis steps 1, 2, and 3 of the raw test score analysis; see Method section), with the statistical design of step 2 compared to step 1 additionally including TIV as covariate. Step 3 compared to step 2 additionally included age-related predictors (i.e., Age, Age quadratic, and the interaction of fs-IQ and Age). At each of the 60 segments of the corpus callosum, the direction and magnitude of the association is visualized by a circle, whereby the size of the circle is proportional to empirical t value and the color (red vs. blue) codes positive and negative associations, respectively. Significance level is adjusted to a False-Discovery-Rate (FDR) of 0.05 whereby for neither v-IQ, p-IQ, nor p-IQ any significant associations were found. Note: the anterior corpus callosum is on the left side of each panel. (JPEG 715 kb)
