# Supplementary figures and images for: The corpus callosum as anatomical marker of intelligence? A critical examination in a large-scale developmental study
Source: Brain Struct Funct. 2017 Aug 11;223(1):285–96. doi: 10.1007/s00429-017-1493-0 (PMC5772147; doi:10.1007/s00429-017-1493-0)

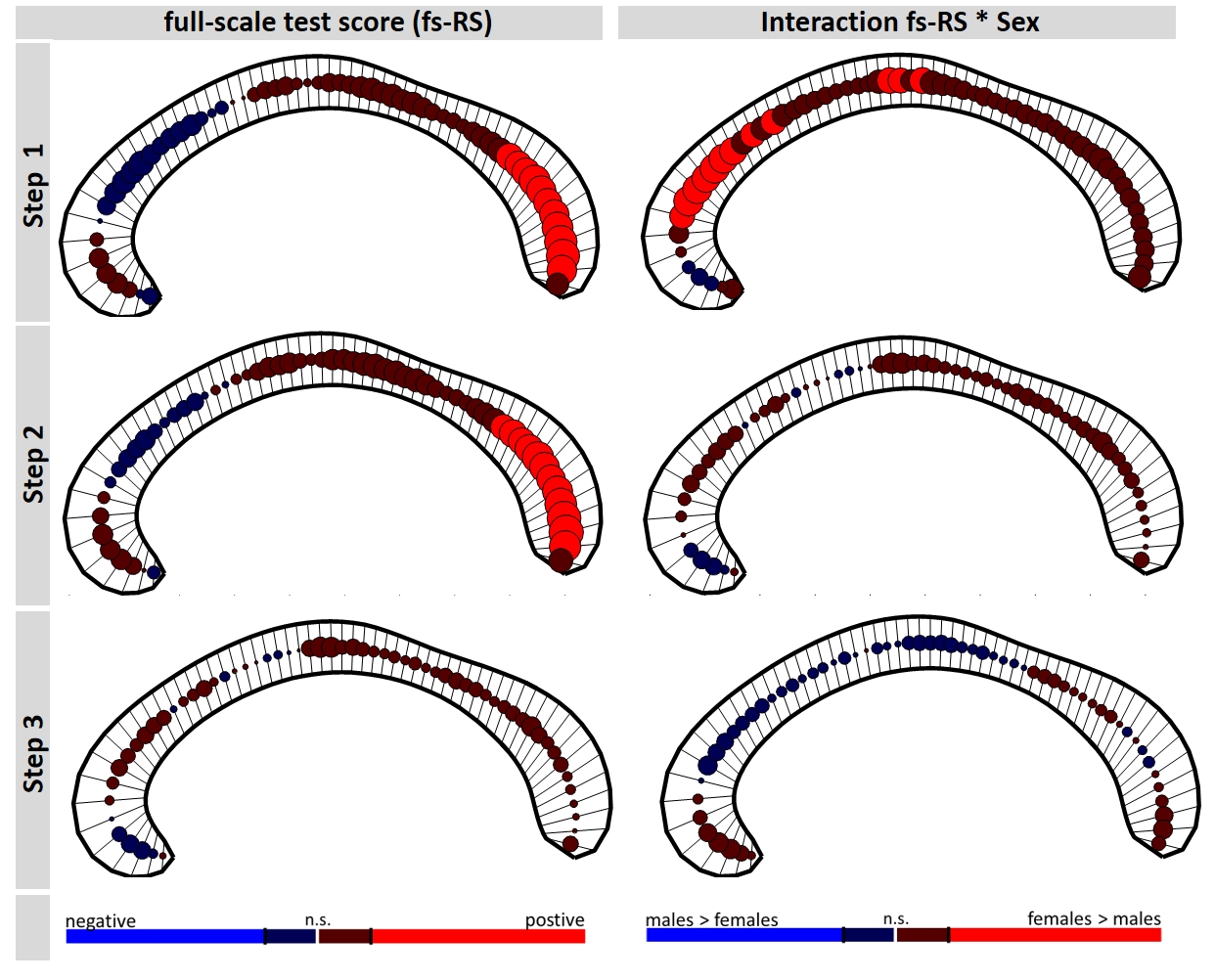

Supplement: Supplementary file 1 — Suppl. Figure 1. Association estimated full-scale raw test score (fs-RS) and of regional callosal thickness (left column) and the interaction of fs-RS with Sex (right column). The rows represent the 3 analysis steps (full model described in Method section), with the statistical design of step 2 compared to step 1 additionally including TIV as covariate, and step 3 compared to step 2 additionally including age-related variables (i.e., Age, Age squared, and the interaction of fs-RS and Age). At each of the 60 segments of the corpus callosum, the direction and magnitude of the association is visualized by a circle, whereby the size of the circle is proportional to empirical t value and the color (red vs. blue) codes positive and negative associations, respectively. Lighter red and lighter blue indicate significant associations, with the significance level is adjusted to a False-Discovery-Rate (FDR) of 0.05. Note: the anterior corpus callosum is on the left side of each panel. (JPEG 410 kb) [file 429_2017_1493_MOESM1_ESM.jpg]

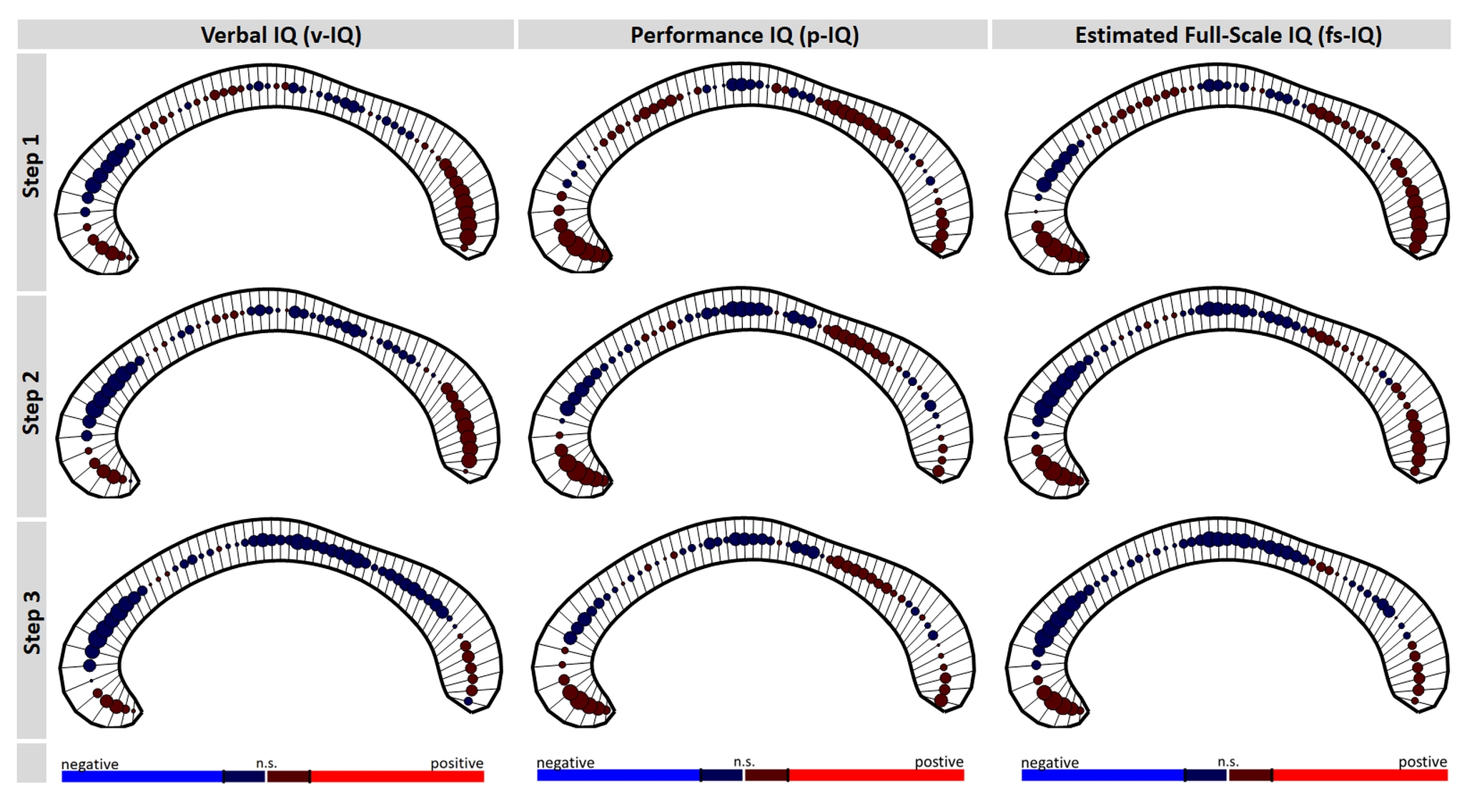

Supplement: Supplementary file 2 — Suppl. Figure 2. Association of verbal (v-IQ, left column), performance (p-IQ, middle column), and estimated full-scale (fs-IQ, right column) deviation IQ with regional callosal thickness. The rows represent three analysis steps (equivalent to analysis steps 1, 2, and 3 of the raw test score analysis; see Method section), with the statistical design of step 2 compared to step 1 additionally including TIV as covariate. Step 3 compared to step 2 additionally included age-related predictors (i.e., Age, Age quadratic, and the interaction of fs-IQ and Age). At each of the 60 segments of the corpus callosum, the direction and magnitude of the association is visualized by a circle, whereby the size of the circle is proportional to empirical t value and the color (red vs. blue) codes positive and negative associations, respectively. Significance level is adjusted to a False-Discovery-Rate (FDR) of 0.05 whereby for neither v-IQ, p-IQ, nor p-IQ any significant associations were found. Note: the anterior corpus callosum is on the left side of each panel. (JPEG 715 kb) [file 429_2017_1493_MOESM2_ESM.jpg]
